# Maternal vaginal fluids play a major role in the colonization of the neonatal intestinal microbiota

**DOI:** 10.3389/fcimb.2023.1065884

**Published:** 2023-03-15

**Authors:** Jingxian Xie, Chen Tang, Shouqiang Hong, Yuntian Xin, Jie Zhang, Yi Lin, Lindong Mao, Yunshan Xiao, Quanfeng Wu, Xueqin Zhang, Heqing Shen

**Affiliations:** ^1^ Department of Obstetrics, Women and Children’s Hospital, School of Medicine, Xiamen University, Xiamen, Fujian, China; ^2^ School of Life Sciences, Xiamen University, Xiamen, Fujian, China; ^3^ State Key Laboratory of Molecular Vaccinology and Molecular Diagnostics, School of Public Health, Xiamen University, Xiamen, Fujian, China; ^4^ Key Laboratory of Urban Environment and Health, Institute of Urban Environment, Chinese Academy of Sciences, Xiamen, Fujian, China

**Keywords:** caesarean section, gut microbiome, newborn, treatment, vaginal fluid, transitional stool

## Abstract

**Background:**

Caesarean section (CS) is associated with newborns’ health risks due to the blocking of microbiome transfer. The gut microbiota of CS-born babies was different from those born vaginally, which may be attributed to reduced exposure to maternal vaginal microbes during labour. To understand the microbial transfer and reduce CS disadvantages, the effect of vaginal microbiota exposure on infant gut microbiota composition was evaluated using 16s rDNA sequencing-based techniques.

**Results:**

Pregnant women were recruited in the Women and Children’s Hospital, School of Medicine, Xiamen University from June 1^st^ to August 15^th^, 2017. Maternal faeces (n = 26), maternal vaginal fluids (n = 26), and neonatal transitional stools (n = 26) were collected, while the participants underwent natural delivery (ND) (n = 6), CS (n = 4) and CS with the intervention of vaginal seedings (I) (n = 16). 26 mothers with the median age 26.50 (25.00-27.25) years showed no substantial clinical differences. The newborns’ gut microbiota altered among ND, CS and I, and clustered into two groups (PERMANOVA *P* = 0.001). Microbial composition of ND babies shared more features with maternal vaginal samples (PERMANOVA *P* = 0.065), while the microbiota structure of ND babies was obviously different from that of sample of maternal faeces. The genus *Bacteroides* in CS-born babies with intervention approached to vaginal-born neonates, compared with CS-born neonates without intervention.

**Conclusions:**

Neonatal gut microbiota was dependent on the delivery mode. And the gut microbiota CS newborns with vaginal seeding shared more features with those of ND babies, which hinted the aberrant gut microbiota composition initiated by CS might be partly mitigated by maternal vaginal microbiota exposure.

## Background

Caesarean **s**ection (CS) is a common obstetric surgical procedure entailing the incision of a woman’s abdomen/uterus to deliver their offspring(s) (Seidu et al., 2020) with the intent to increase the chances of successful childbirth and to protect the life security of both the mother and the newborn (Betran et al., 2016; Cegolon et al., 2020). Over the past two to three decades, global CS surgery rates have been growing steadily but rapidly for women of all ages, races, and medical conditions. According to the World Health Organization (WHO), an estimated 21.1% of births occurred by CS in 2015, which was almost twice than that in 2000 (12.1%), and the rates were even higher in certain developed countries and regions (Ekstrom et al., 2020; Betran et al., 2018; Boerma et al., 2018; Wells et al., 2019). Similar patterns could also be observed in China, in which 28.8% and 34.9% of babies were delivered by CS in 2008 and 2014, respectively (Li et al., 2017).

However, prior publications have demonstrated that CS was associated with adverse short- or long-term effects on newborns, including the dysplasia of the immune system, infections, allergies, and inflammatory disorders (Mueller et al., 2015a; Mueller et al., 2017a; Rusconi et al., 2017). The conventional view concerning this correlation was that CS newborns were subjected to different hormonal, physical (mechanical forces), bacterial, and therapeutic conditions (Sandall et al., 2018). Among these conditions, differences in microbial colonization induced by delivery mode were thought to be one of the determining factors (Martin et al., 2016; Mueller et al., 2017a). The gut microbiota has been increasingly recognized as an important contributor to human health, especially for infants, whose maturity of immune system and overall physiology are influenced the gut microbiota (Korpela et al., 2018). Altered colonization of the gut microbiota in CS-born babies may partially account for the increased risk of adverse health conditions (Butler É et al., 2020). The sterility of the womb was widely accepted for many years (Perez-Muñoz et al., 2017). Although it is controversial about whether the presence of bacterial DNA contradicts the “sterile womb paradigm”, it does not demonstrate the presence of a living microbiota (Ferretti et al., 2018; He et al., 2020). The prevailing view held that human fetal environment is sterile and the neonate’s microbiome is acquired during and after birth (Perez-Muñoz et al., 2017; Walter and Hornef, 2021). Fetal development is an important period for human beings, and the extent to which modern practices, like CS, alter the microbial composition are still not completely understood (Dominguez-Bello et al., 2019). The exposure of newborns to the maternal vaginal microbiota might be interrupted by CS. Unlikely to vaginally delivered newborns, who are first exposed to a wide array of microbes during labour *via* direct contact with the birth canal, CS newborns are initially contacted with microbes from the delivery room and the mothers’ skin (Chu et al., 2017). In addition, prophylactic antibiotics administered before or during caesarean surgery may also result in the failure of newborns to acquire the normal microbial inoculum (Azad et al., 2016; Stearns et al., 2017). Thus, vaginal fluids exposure (also known as “vaginal seedings”) on CS-born babies shortly after birth is a potential way for reducing differences in the gut microbiota between CS-born and vaginal-born neonates (Butler É et al., 2020; The American College of Obstetricians and Gynecologists, 2017). A better understanding about such practice on infant gut microbiota composition are required to break this cycle. Moreover, exploring an effective intervention measure which is simple, convenient, and cost effective to compensate for the differences in gut microbiota composition of CS births could be a prospective application. Here, we used transitional stool as the biological sample in our present work, which was defined as the faeces excreted by newborns between 36 h and 72 h after birth (Mueller et al., 2017b). It represents the transitional state of meconium and may vary quickly *via* early-life microbial colonization in the surrounding environment. Only a few studies have aimed to explore the microbial community in transitional faeces.

Via the swabbing of CS newborns with their mothers’ vaginal fluids, we aimed to explore the effect of vaginal microbiota exposure on infant gut microbiota composition in this study. By analyzing the pros and cons of the treatment through a population-based intervention study in Xiamen, PR China, we assess its effectiveness and performance in altering the microbes in newborns’ transitional stools.

## Methods

### The recruitment of subjects

This intervention study invited pregnant women who received antenatal care at the Women and Children’s Hospital, School of Medicine, Xiamen University, from June 1^st^, 2017 to August 15^th^, 2017. The inclusion criteria were briefly listed as follows: 1) gestational age between 37 and 42 weeks; 2) pregnant women who had regular prenatal visits and for whom all clinical data could be obtained; and 3) newborns who were full-term deliveries. Pregnant women with the following complications were excluded: 1) infectious diseases caused by bacteria, viruses, or parasites; 2) inflammatory diseases (e.g., ankylosing spondylitis); 3) metabolic diseases such as diabetes mellitus; 4) pregnancy-associated illnesses including preeclampsia and gestational hypertension; 5) reproductive system disorders (e.g., an ovarian cyst); 6) abnormal pregnancy state (e.g., preterm birth); 7) genetic diseases such as thalassemia; and 8) tumors, including pituitary adenomas and uterine fibroids. The specific analytical protocol of this study is shown in **Figure S1**. The basic information of the pregnant were collected through the last prenatal health checkup and lifestyle questionnaires.

The protocol of our present research was approved by the Medical Ethics Committee of the Women and Children’s Hospital, School of Medicine, Xiamen University (KY-2018-020). All procedures were conducted in accordance with the Declaration of Helsinki. All patients were required to provide written informed consent prior to participation. No adverse events were reported for any of the newborns in this study.

### Treatment and sample collection

Pregnant women scheduled to have a CS surgery were administrated with prophylactic antibiotics 15 to 60 minutes before skin incision and divided into the two groups according to their willingness to have their newborns swabbed with the vaginal fluids. Before delivery (CS or natural delivery), a 7 × 5 cm four-layered piece of gauze was double-folded and soaked in sterile saline and then inserted into the lower vagina for at least 30 mins before the administration of prophylactic antibiotics, and then removed and kept at room temperature in a sterile collector. The gauze was then divided vertically into two equal parts. One part of the gauze was temporally stored in a 50 mL microcentrifuge tube for subsequent analyses of microbiome, while the other part was used to treat CS newborns (n = 16) as soon as they were born. The principle of ‘center-to-periphery’ was strictly followed during the swabbing procedure, which began on the lips, followed by the face, the thorax, the arms, the legs, and finally the back; the complete process took approximately 30 s for each newborn. Six vaginal-born and four CS-born babies were swabbed with gauze containing sterile saline solution and were used as positive or negative references. All treatments were performed in the delivery room. Transitional stool was first collected by sterile diapers between 36 h and 72 h after birth, and then 1-2 g of the sample was transferred into a 50 mL microcentrifuge tube. Approximately 1-2 g of fresh maternal faeces was collected into a 50 mL microcentrifuge tube at the last excretion before delivery. All samples were placed into containers of ice and transported to the laboratory within 1 h after collection and stored at -80°C for long-term storage until further processing.

### Microbial DNA extraction and 16S rDNA amplification

Microbial DNA in all samples was extracted utilizing the MoBio Powersoil DNA Isolation Kit (QIAGEN, German) according to the manufacturer’s instructions. A NanoDrop ND-1000 spectrophotometer (NanoDrop Technologies, USA) was used to determine the DNA concentration. The extracted DNA was stored at -20°C until further analysis. The hypervariable V3-V4 regions of the 16S rDNA gene were amplified with the specific primers 341F (5’-CCTACGGGNGGCWGCAG-3’) and 806R (5’-GGACTACHVGGGTATCTAAT-3’). PCRs were performed in triplicate in a 50 μL mixture containing 5 μL of KOD buffer (10 ×), 5 μL of dNTPs (2 mM), 3 μL of MgSO_4_ (25 mM), 1.5 μL of 341F/806R primer (10 μM), 1 μL of KOD Polymerase, and 100 ng of template DNA. The PCR reagents were purchased from TOYOBO, Japan, and the PCR protocol was carried out for 30 cycles using the following parameters: 94°C pre-denaturation for 2 min, 98°C denaturation for 10 s, 50°C annealing for 30 s, 68°C annealing for 30 s, and hold at 4°C. After amplification, all of the PCR products were pooled, purified, and quantified using the AxyPrep DNA Gel Extraction Kit (Axygen Biosciences, USA) and the ABI Step-One-Plus Real-Time PCR System (Life Technologies, USA) according to the standard protocols.

### 16S rDNA sequencing and data processing

The microbiome profiles were analyzed by 16S rRNA gene amplicon sequencing with the Illumina MiSeq 250PE platform (Illumina, San Diego, CA, USA). USEARCH software (version 8.1.1861) was applied to turn paired-end sequencing reads into merged, denoised, chimera-free, inferred sample sequences, and the sequence processing steps are described below in detail. Raw sequencing reads were filtered using FASTP software (version 0.18.0) to remove adapters and low-quality reads that would affect the subsequent assembly and analysis to obtain clean high-quality paired-end reads. Paired-end reads were merged as raw tags using FLASH (version 1.2.11), with a minimum overlap of 10 bp and mismatch error rates of 20% (Magoč and Salzberg, 2011), and further merged as raw amplicon sequence variants (ASVs) with a minimum overlap of 10 bp. Noisy sequences of raw tags were filtered under specific filtering conditions to obtain clean tags. The high-quality clean tags were clustered into OTUs of ≥ 97% similarity using the UPARSE (version 9.2.64) pipeline. All chimeric tags were removed using the UCHIME algorithm, and effective tags were finally obtained for the next analysis step. The tag sequence with the highest abundance was selected as the representative sequence within each cluster. The representative OTU sequences were classified into organisms by a naive Bayesian model using the RDP classifier (version 2.2) (Wang et al., 2007) based on the SILVA database (version 132) (Pruesse et al., 2007).

### Statistical and bioinformatic analyses

Because the number of subjects was less than 50, the Shapiro-Wilk test and Levene’s test were performed to assess the normality of distributions and the homogeneity of variance, respectively. One-way analysis of variance (ANOVA) was performed to compare normally distributed continuous variables, while the Kruskal-Wallis H test was conducted to compare unevenly distributed variables. Comparisons of Alpha and Beta diversity between any two groups were performed by utilizing Welch’s t test, whereas Kruskal-Wallis H test was used for the comparison among groups. Statistical analysis of the clinical data was achieved by Statistic Package for Social Science software (SPSS) (SPSS Inc., USA) (version 26.0). P < 0.05 was considered to be statistically significant.

Alpha-diversity was assessed by the observed species (Sobs) index, inverse Simpson index, Shannon index, and Pielou evenness index, while beta-diversity was calculated by the Bray-Curtis distance and illustrated by NMDS analysis. Alpha- and beta-diversity, as well as the NMDS were generated in the R project Vegan package (version 2.5.3) (Dixon, 2003) and plotted in the R project ggplot2 package (version 2.2.1) (Wickham et al., 2016). Circular layout representations of species abundance were graphed using circos (version 0.69-3) (Krzywinski et al., 2009). Between groups, Venn analysis was performed in the R project VennDiagram package (version 1.6.16) (Chen and Boutros, 2011). A ternary plot of taxa abundance was plotted using the R ggtern package (version 3.1.0) (Hamilton and Ferry, 2018).

## Results

### Baseline clinical information of pregnant women

A total of 111 mother-newborn dyads were initially recruited. As shown in **Figure S2**, we excluded 85 pairs due to the lack of integrated bio-samples (n = 69), health check-up data (n = 9), and self-reported diseases information (n = 7). Finally, 26 mother-newborn dyads were ultimately included according to our inclusion and exclusion criteria, and they provided 78 samples for the subsequent analyses. Missing values of age (n = 2), enrolment age (n = 1), haemoglobin (n = 1), alanine aminotransferase (ALT) (n = 1), aspartate aminotransferase (AST) (n = 1), serum total bilirubin (STB) (n = 1), and serum conjugated bilirubin (SCB) (n = 1) were interpolated using the multiple imputation method. The basic information of the pregnant women is shown in **Table 1**. The median maternal age was 26.50 (25.00-27.25) years, and the average body mass index (BMI) was 20.82 ± 2.45. No participants in the present research subjected active or passive smoking. Other demographic and clinical indicators among groups were roughly similar except for spouse age (SA) and AST (ANOVA, *P*_SA_=0.033; Kruskal-Wallis H test, *P*_AST_=0.022). Despite a significant difference in AST, other maternal biological factors were well homogenized among the different groups. In combination with the clinical indices, participants in this study were overall of a relatively good health status, and the maternal clinical statuses were comparable.

**Table 1:** Maternal Clinical Information at Baseline.

Variables	Overall	VD	CS	I	*P* value
	N=26	n=6 (23.08%)	n=4 (15.38%)	n=16 (61.54%)	
Marriage Age (years)	26.50 (25.00-27.25)	26.00 (25.75-31.15)	25.25 ± 1.11	26.16 ± 0.70	0.707
Enrollment Age (years)	30.00 (27.00-32.00)	26.67 ± 1.33	33.25 ± 1.97	30.00 (28.55-32.00)	0.055
Spouse Age (years)	32.08 ± 5.52	26.98 ± 2.51	34.00 (34.00-40.00)	33.00 ± 1.11	0.033
Age of Menarche (years)	14.52 ± 1.68	14.00 ± 0.68	15.75 ± 0.75	14.41 ± 0.42	0.255
SBP (mmHg)	103.04 ± 9.91	105.17 ± 3.44	109.25 ± 5.31	100.69 ± 2.49	0.262
DBP (mmHg)	64.40 ± 7.62	65.33 ± 3.03	68.00 ± 4.97	63.15 ± 1.82	0.512
Height (cm)	158.82 ± 5.14	161.08 ± 2.27	160.50 ± 2.99	157.55 ± 1.18	0.288
Weight (Kg)	53.82 ± 7.91	53.35 ± 1.46	53.50 ± 4.94	54.08 ± 2.24	0.979
BMI (kg/m^2^)	20.82 ± 2.45	20.03 ± 0.31	20.18 ± 1.28	21.27 ± 0.70	0.507
Hemoglobin (g/L)	122.60 ± 8.80	125.83 ± 3.04	114.00 (113.00-121.75)	122.98 ± 2.37	0.208
Leukocyte (×10^9^/L)	7.08 (6.50-8.01)	7.86 ± 0.75	5.95 (5.18-7.10)	7.29 ± 0.27	0.155
Platelet (×10^9^/L)	230.52 ± 41.64	232.50 ± 11.68	214.50 ± 4.13	233.78 ± 12.55	0.644
Blood Glucose (mmol/L)	4.82 ± 0.31	4.74 ± 1.60	4.79 ± 0.18	4.86 ± 0.07	0.720
ALT (U/L)	13.30 (10.75-18.02)	11.00 ± 1.02	39.52 ± 16.21	15.34 ± 1.33	0.120
AST (U/L)	16.90 (14.00-19.00)	14.18 ± 0.71	22.00 ± 2.86	17.34 ± 1.01	0.022
Albumin (g/L)	42.63 ± 5.78	44.03 ± 1.74	40.60 ± 1.64	41.55 (38.83-44.05)	0.350
STB (μmol/L)	10.82 ± 2.73	10.44 ± 0.97	7.10 (6.63-10.50)	11.64 ± 0.64	0.091
SCB (μmol/L)	3.59 ± 1.54	3.52 ± 0.52	2.91 ± 0.95	3.78 ± 0.40	0.613
SCr (μmol/L)	56.50 ± 11.18	58.05 ± 5.00	50.48 ± 3.80	57.43 ± 2.91	0.518
BUN (mmol/L)	3.07 ± 0.74	3.11 ± 0.24	2.87 ± 0.45	3.10 ± 0.20	0.856
Smoking (n, %)	0 (0)	0 (0)	0 (0)	0 (0)	–
Use of Antibiotic before Delivery (n, %)	0 (0)	0 (0)	0 (0)	0 (0)	–
Use of Prebiotics/Probiotics (n, %)	0 (0)	0 (0)	0 (0)	0 (0)	–

Shapiro-Wilk tests were performed to test the assumptions of normality. Normally distributed variables with even variance were presented as mean ± SD, skewed variables as median (lower quartile to upper quartile), and categorical variables as n (%). Continuous variables were compared using ANOVA or Kruskal-Wallis test depending on distribution.

VD, Pregnant women underwent vaginal delivery; CS, Pregnant women underwent cesarean delivery; I, Pregnant women underwent cesarean delivery with vaginal fluid swabbing intervention on their newborns; SBP, Systolic Blood Pressure; DBP, Diastolic Blood Pressure; BMI, Body mass index; ALT, Alanine aminotransferase; AST, Aspartate aminotransferase; STB, Serum total bilirubin; SCB, Serum conjugated bilirubin; SCr, Serum creatinine; BUN, Blood Urea Nitrogen. No pregnant woman reported tobacco consumption, and the use of antibiotics as well as prebiotics or probiotics. Therefore, no statistical tests were performed. And the P values were displayed as '-'.

### Microbiome overview of the maternal and neonatal samples

The diversity of the microbiota in a given habitat reflects the composition and relative abundance of the community. Approximately 460 bp of PCR products were generated by amplifying the V3-V4 region of the 16S rRNA gene to compare the bacterial diversity among samples. DNA sequencing after quality filtering yielded 8.29 million paired-end reads, which further merged into 7.21 million tags, with a minimum of 64253 tags per sample (average of 92448 ± 8822), and ultimately formed 27801 operational taxonomic units (OTUs).

**Figure 1:**
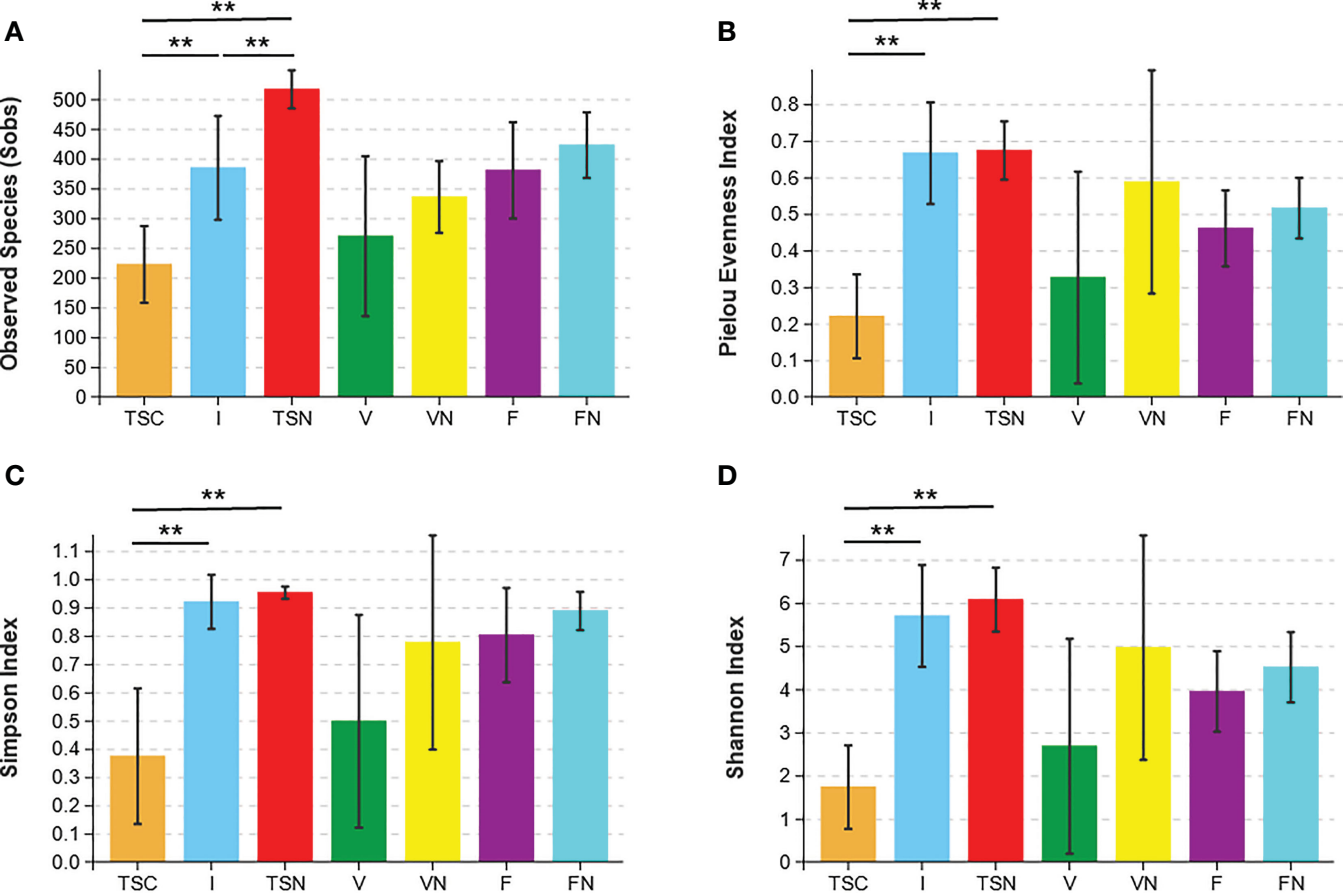
Microbial alpha diversity indices. The ecological diversity of microbiota in transitional stools of newborns (36-72h after birth), virginal fluids (before delivery) and stool of mothers (the last excretion before delivery) was measured by Sobs **(A)**, Pielou evenness index **(B)**, the Simpson index **(C)**, and the Shannon index **(D)**. The *P* values were conducted by Welch’s t test. Statistical significance is displayed as *P<0.05 and **P<0.01. Sobs, Observed species; TSC, Transitional stools of caesarean delivered neonates; I, Transitional stools of caesarean delivered neonates with the treatment of swabbing maternal vaginal fluid; TSN, Transitional stools of natural delivered neonates; V, Vaginal fluids of the pregnant women who underwent caesarean delivery; VN, Vaginal fluids of the pregnant women who underwent natural delivery; F, Feces of the pregnant women who underwent caesarean delivery; FN, Feces of the women who underwent natural delivery. Symbol "*" was presented above each plot.

Rarefaction curves evaluated the OTU richness and represented whether a reasonable sampling size (sequencing depth) was used. As shown in **Figure S3**, the almost horizontal asymptotic curves indicated that the sequencing depth was sufficient for our research. The values of observed species (Sobs), Pielou evenness, Shannon, and inverse Simpson indices were calculated to thoroughly assess alpha-diversity. The Sobs index indicated the actual detected OTUs, while the Pielou evenness index referred to the species equitability within each group. The Simpson and Shannon indices measured the degree of species asynchrony and stability, and higher values represented higher richness, evenness, or both (Luo et al., 2017). As shown in **Figure 1A**, the highest Sobs value could be observed in the transitional **s**tool samples of **n**aturally delivered newborns (TSN), and the OTUs differed substantially between the transitional **s**tool samples of **c**aesarean-section newborns (TSC) and the TSN, which demonstrated that CS surgery reduced the actual number of species of newborn gut microbes compared with vaginal delivery. In contrast, the TSC group presented the lowest evenness according to the value of the Pielou evenness index (**Figure 1B**) and there was a significant difference between the TSC and TSN groups (Welch’s t test, *P _Pielou_* =0.001). The above analyses revealed that CS surgery led to an obvious alteration in bacterial richness and evenness. Good agreement was exhibited in the Simpson index and Shannon index, which suggested that the gut microbial composition of CS newborns was less diverse (Welch’s t test, *P _Simpson_* =0.017, *P _Shannon_* <0.001), while a lesser degree of dominant bacteria and distribution homogeneity were observed in CS newborns with swabbing vaginal fluid **i**ntervention (I) (**Figures 1C, D**). The aggregate analyses of alpha-diversity indicated that the neonatal gut microbiota was affected by both the mode of delivery and the swabbing treatment. Beyond that, pregnant women who underwent different labour modes showed no significant differences in intestinal (Welch’s t test, *P _Sobs_* =0.169, *P _Pielou_* =0.207, *P _Simpson_* =0.081, *P _Shannon_* =0.186) and vaginal fluid microbiota (Welch’s t test, *P _Sobs_* =0.152, *P _Pielou_* =0.188, *P _Simpson_* =0.246, *P _Shannon_* =0.178).

**Figure 2:**
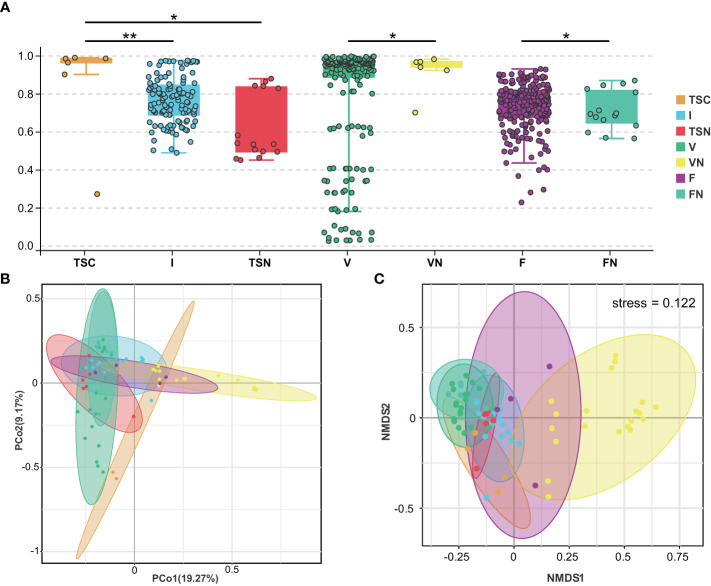
Visualization of beta-diversity index **(A)**, PcoA **(B)**, and NMDS **(C)**. These plots were conducted basing on Bray-Curtis Distance. Each dot represents one sample. In **(A)**, mean beta-diversity (distance from centroid) ± standard error.Statistical significance is displayed as *P < 0.05 and **P < 0.01. TSC, Transitional stools of caesarean delivered neonates; I, Transitional stools of caesarean delivered neonates with the treatment of swabbing maternal vaginal fluid intervention; TSN, Transitional stools of natural delivered neonates; VN, Vaginal fluids of the pregnant women who underwent natural delivery; V, Vaginal fluids of the pregnant women who underwent caesarean delivery; FN, Feces of the women who underwent natural delivery; F, Feces of the pregnant women who underwent caesarean delivery.

We further investigated beta-diversity according to the Bray-Curtis distance to compare the microbial community structures among groups, as this method provided a model to describe the overall pattern of community composition based on OTUs. Interestingly, some results seem to be inconsistent with those obtained from alpha-diversity analyses (**Figure 2A**). Briefly, significant differences could be observed between the neonates of CS and I group (Adonis test, *P*= 0.001), as well as the CS and vaginally delivered newborns (Adonis test, *P* = 0.011). The gut microbiota of CS with vaginal seedings and vaginally delivered newborns seemed to show no substantial difference in terms of beta-diversity. The above-mentioned results hinted the microbial composition of neonates was highly dependent on the mode of delivery. Besides, the microbiome of vaginal fluids and stools samples were different between pregnant women who underwent different labour modes (Adonis test, *P _V-VN_* =0.034, *P _F-FN_* =0.047). Further, the visualization of principal coordinate analysis (PcoA) and non-metric multidimensional scaling (NMDS) analysis based on the Bray-Curtis distance all displayed clear ordinations that indicate the gut microbes of the CS newborns with swabbing treatment were more similar with those of the vaginally delivered newborns, rather than CS babies (**Figures 2B, C**). The above-mentioned results concluded that the infant gut microbiota shared more features with maternal vaginal fluids, and the microbial composition of transitional stool samples in CS-born babies with vaginal seedings tended to be more similar to that of TSN samples. The detailed information of statistical analyses was shown in **Table 2**.

**Table 2:** Statistical analyses of beta-diversity.

Groups	*df*	F value	R^2^	*P* value
F-vs-FN	1	1.784	0.069	0.056
V-vs-VN	1	2.207	0.091	0.043
F-vs-V	1	11.85	0.24	0.001
FN-vs-VN	1	2.11	0.21	0.004
TSC-vs-TSN	1	2.683	0.251	0.006
TSC-vs-I	1	2.814	0.135	0.004
TSN-vs-I	1	1.432	0.067	0.083
TSC-vs-F	1	2.692	0.109	0.007
I-vs-F	1	5.103	0.131	0.001
TSC-vs-V	1	3.151	0.125	0.001
I-vs-V	1	8.263	0.196	0.001
TSN-vs-FN	1	3.037	0.233	0.005
TSN-vs-VN	1	1.483	0.156	0.065

df, degree of freedom; TSC, Transitional stools of caesarean delivered neonates; I, Transitional stools of caesarean delivered neonates with the intervention of swabbing maternal vaginal fluids; TSN, Transitional stools of natural delivered neonates; VN, Vaginal fluids of the pregnant women who underwent natural delivery; V, Vaginal fluids of the pregnant women who underwent caesarean delivery; FN, Feces of the women who underwent natural delivery; F, Feces of the pregnant women who underwent caesarean delivery.

### The variation in microbiota caused by delivery modes and treatment

**Figure 3:**
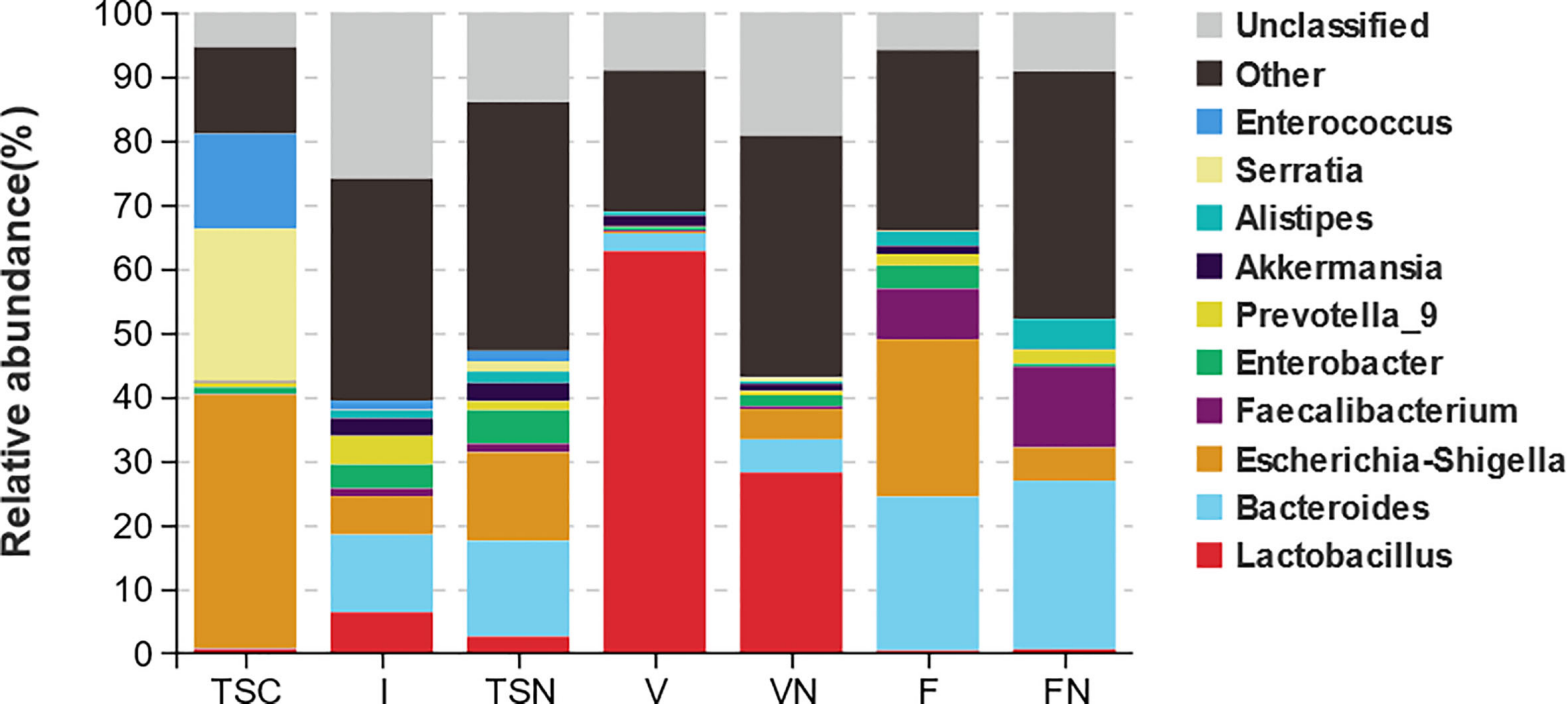
Taxa distribution plots at phylum and genus level. The relative abundances of microbial communities at genus level. TSC, Transitional stools of caesarean delivered neonates; I, Transitional stools of caesarean delivered neonates with the treatment of swabbing maternal vaginal fluid intervention; TSN, Transitional stools of natural delivered neonates; VN, Vaginal fluids of the pregnant women who underwent natural delivery; V, Vaginal fluids of the pregnant women who underwent caesarean delivery; FN, Feces of the women who underwent natural delivery; F, Feces of the pregnant women who underwent caesarean delivery.

**Figure 4:**
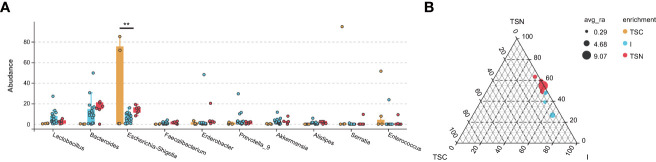
The Effect Analyses of Swabbing Exposure. The characteristic taxa were measured by indicator analysis **(A)** and ternary plot **(B)**. The P values were conducted by Tukey HSD test. Statistical significance is displayed as *P < 0.05 and **P < 0.01. TSC, Transitional stools of caesarean delivered neonates; I, Transitional stools of caesarean delivered neonates with the treatment of swabbing maternal vaginal fluid intervention; TSN, Transitional stools of natural delivered neonates; VN, Vaginal fluids of the pregnant women who underwent natural delivery; V, Vaginal fluids of the pregnant women who underwent caesarean delivery; FN, Feces of the women who underwent natural delivery; F, Feces of the pregnant women who underwent caesarean delivery.

The analysis at the genus level indicated the microbial community of CS newborns consisted primarily of *Bacteroides* (12.10%), *Lactobacillus* (6.38%), and *Escherichia-Shigella* (6.03%) after the vaginal seedings intervention (**Table 3**; **Figures 3**, **4**), which was more closed to vaginally delivered babies. Among them, the genus of *Bacteroides* tended to be similar to those of vaginally delivered newborns (14.95%), which was significantly higher than CS-born neonates (0.15%). Statistical analysis further indicated that no obvious restoration pattern existed at the genus level, although the genus of *Faecalibacterium*, *Enterobacter*, and *Akkermansia* presented such trends (**Table 3**).

**Table 3:** Relative abundance at the genus level.

Relative abundance (%)	TSC	I	TSN	F	FN	V	VN
*Lactobacillus*	0.68	6.38	2.57	0.43	0.62	62.71	28.20
*Bacteroides*	0.15	12.10	14.95	23.95	26.26	2.77	5.12
*Escherichia-Shigella*	39.49	6.03	13.80	24.57	5.19	0.23	4.75
*Faecalibacterium*	0.10	1.19	1.34	7.88	12.59	0.24	0.45
*Enterobacter*	1.03	3.72	5.23	3.65	0.47	0.51	1.71
*Prevotella_9*	0.72	4.49	1.38	1.80	2.21	0.12	0.64
*Akkermansia*	0.25	2.72	2.87	1.26	0.10	1.67	1.14
*Alistipes*	0.00	1.19	1.77	2.23	4.69	0.59	0.37
*Serratia*	23.80	0.09	1.62	0.08	0.00	0.03	0.60
*Enterococcus*	14.81	1.50	1.63	0.02	0.00	0.02	0.03
Other	13.54	34.65	38.79	28.24	38.65	22.02	37.74
Unclassified	5.44	25.95	14.05	5.90	9.22	9.09	19.25

TSC, Transitional stools of caesarean delivered neonates; I, Transitional stools of caesarean delivered neonates with the intervention of swabbing maternal vaginal fluid; TSN, Transitional stools of natural delivered neonates; VN, Vaginal fluids of the pregnant women who underwent natural delivery; V, Vaginal fluids of the pregnant women who underwent caesarean delivery; FN, Feces of the women who underwent natural delivery; F, Feces of the pregnant women who underwent caesarean delivery.

**Figure 5:**
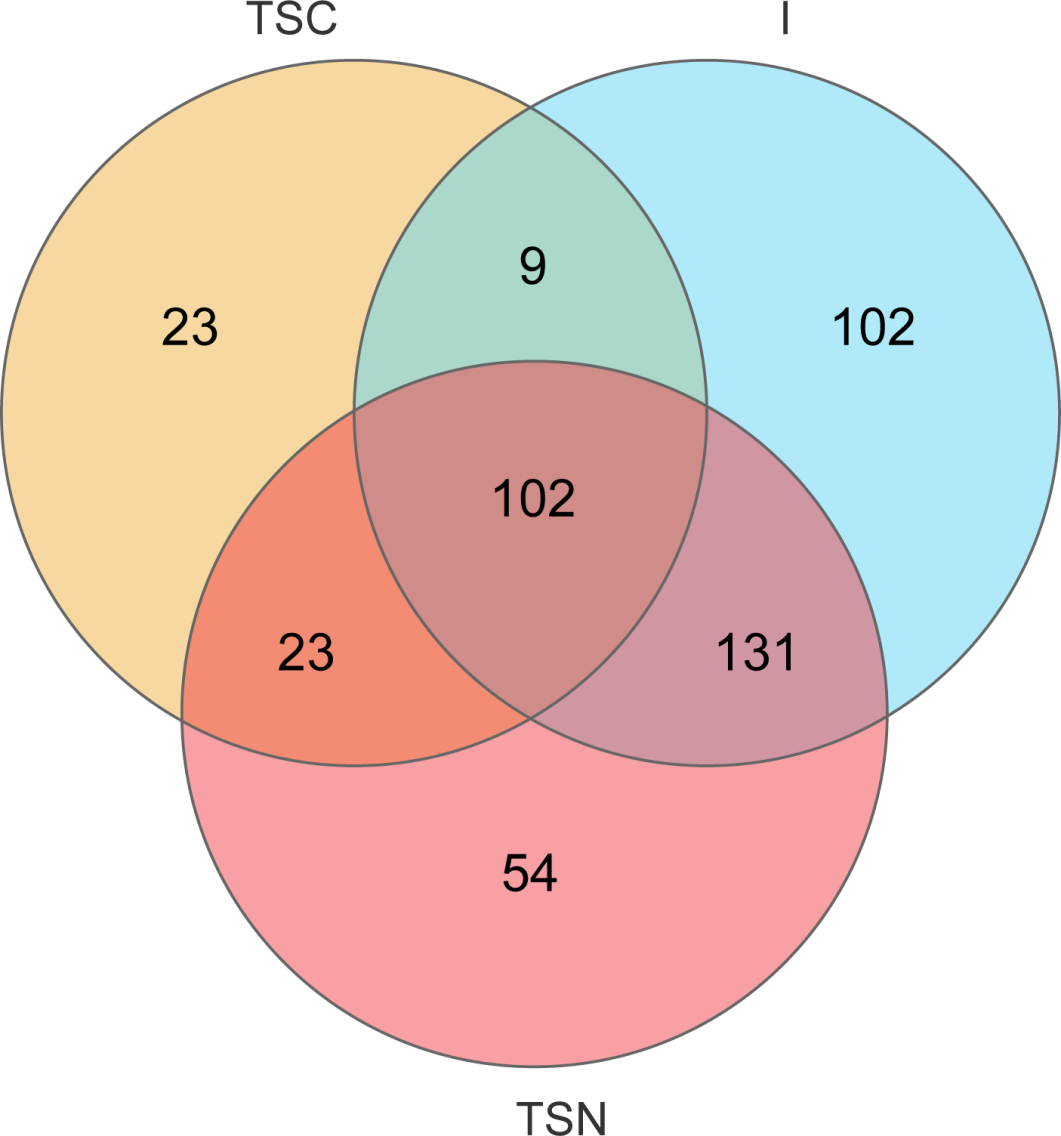
VENN diagrams at genus level. VENN diagram was used to represented the common and unique genera among different groups. TSC, Transitional stools of caesarean delivered neonates; I, Transitional stools of caesarean delivered neonates with the treatment of swabbing maternal vaginal fluid; V, Vaginal fluids of the pregnant women who underwent caesarean delivery.

The VENN plots confirmed the above results from another aspect, which more shared taxa were detected between vaginally delivered and CS newborns in the intervention group, than those with CS newborns (**Figure 5**).

## Discussion

In this population-based intervention study, the notion that neonatal microbial composition was dependent on the mode of delivery was confirmed by our results, which were in good agreement with previous researches (Fouhy et al., 2019). In addition, we observed the presence of restoration pattern of microbial composition at the genus level. It is a potential method of neonatal exposure to maternal vaginal microbiota shortly after CS birth to promote the development of the gut microbiome, which has gained traction in recent years (Obstetricians and Gynecologists, 2017). However, very few studies have focused on the microbiota in transitional stool to date. A pilot study conducted by Mueller et al. showed that significantly lower relative abundances of the genera *Bacteroidetes*, *Parabacteroides*, and *Clostridium* were observed in CS-born babies (Mueller et al., 2017b). In our present work, *Bacteroides* exhibited restoration pattern after vaginal seedings, while no other observed differences between the both CS groups. According to previous studies, the gut microbiome in CS-born infants might not be altered *via* vaginal seedings. Wayne S. Cutfield et al. assessed the effect of oral administration of maternal vaginal microbes to restore gut microbial composition among CS-born infants. However, there were no observed differences in gut microbiome composition between CS-born infants with or without the intervention at 1 month or 3 months after birth. And CS-born infants displayed the characteristic signature of low *Bacteroides* abundance compared with vaginal-born ones (Wilson et al., 2021). Our results may not align completely with others, which might be attributed to the dissimilarity in the collection, storage processing or analytic platforms used in the different studies (Perez-Muñoz et al., 2017). Maternal fecal microbiota transplantation (FMT) appears to be a more promising strategy. Willem M. de Vos et al. designed a proof-of-concept study that verified the gut microbial development could be restored rapidly *via* FMT (Korpela et al., 2020). Meanwhile, this study also demonstrated the neonatal gut microbiota is highly dependent on the delivery mode, which were in general agreement with our conclusions.

Interestingly, the microbiome appeared to show significant discrepancies in the vaginal fluid samples of pregnant women who were subjected to different labour modes. This conclusion was consistent with Marta Selma-Royo et al. (Selma-Royo et al., 2020) and Romero R et al. (Romero et al., 2014), which described the variations occurred during gestation, mainly in the intestinal and vaginal microbiomes. These studies also indicated that delivery mode significantly affected the maternal microbiota composition at delivery. One of the probable reasons was that medical decisions for different delivery modes depended on maternal health states and might be directly reflected in the vaginal microbiota. Moreover, previous investigations have proposed that the use of antibiotics during the gestation or the periparturient period would also cause those differences (Mueller et al., 2015b). Early studies observed that the abundance of the phylum Bacteroidetes participated in modulating the weight development of newborns (Ley et al., 2006; Turnbaugh et al., 2006). Additionally, it has been well documented that an increase in the abundance of the phylum *Proteobacteria* was associated with onset risks of diabetes and obesity (Su et al., 2018). In our present work, the increased richness of the phyla Bacteroidetes and the decreased richness of Proteobacteria might exert helpful impacts on the proper development of babies. At the genus level, the reduced abundance of pernicious bacteria *Escherichia-Shigella* was considered to be beneficial to CS neonates. Taken together, these findings indicate that the finer targeting of the vaginal fluids before exposure would determine the extent to which this intervention method could be popularized. The microbiome of transitional faeces reflects the colonization of microorganisms *in utero* as well as under the influence of external environmental factors shortly after birth (Mueller et al., 2017b). In this research, the gut bacterial composition of vaginally born babies was similar to that of the maternal vaginal fluid, but similar results could not be observed in CS newborns without treatment, while vertical transmission might be achieved by swabbing treatment among CS newborns. This phenomenon has aroused our concern. It is widely accepted that the effects of the natural delivery process are comprehensive, including physical, chemical, and biological effects, which generally last for one to two hours, covering the whole period of the second stage of labour (Sandall et al., 2018). During childbirth, newborns may inhale (swallow) the vaginal contents in their mother’s birth canal (Butler É et al., 2020). The stomachs of newborns are pH neutral for several hours post birth as a result of swallowing the amniotic fluid *in utero*, thereby enabling the survival of ingested bacteria (Avery et al., 1966). However, the exposure application in our work deliberately avoided contact with the CS newborns’ oral cavity, which might eliminate microbial colonization *via* the oral transmission route. Hence, the restoration by swabbing vaginal fluids may be inefficient due to incomplete biological effects and the absence of chemical and physical effects. On the other hand, antibiotic therapy is a common preventive measure to avoid intra- or post-surgical infections. Nevertheless, residual concentrations of antibiotics in maternal blood might act on the newborn through circulation and last for a period of time post birth, which also means that the microorganisms in the vaginal secretions may not fully colonize the newborn because of the influence of the residual antibiotics.

## Strengths and limitations

### Strengths

The main strength is that our present study is the first research to our best knowledge focusing on the microbial transfer and the intervention programs for CS-born neonates using the samples of transitional stools among Chinese population. The other strength is that the refined processes of sampling and experimental manipulations made the results more reliable and accurate.

### Limitations

There were several main limitations in our research. First, due to the difficulty in sampling, the number of subjects in CS group was relatively small, potentially leading to misinterpretation. Second, this study was based on cross-sectional data, the dynamic changes of microbiome could not be observed, which limited the generalizability of this research.

## Conclusion

Through the intervention project conducted in the Chinese population, we first verified the microbial alterations induced by different delivery modes, including noticeable changes in alpha- and beta-diversity, the structure of gastrointestinal bacterial communities. Second, the issue of the source of gut microbiota for newborns born *via* different delivery modes was partly resolved according to NMDS analysis. The structure of neonatal gut microbes shared more features with maternal vaginal samples among vaginally delivered babies. But such phenomenon could not be found among CS newborns without vaginal seedings. Last through ternary plots and Venn diagrams, we concluded that swabbing exposure could partly restored the dysbiosis of gut microbiota caused by CS.

## Data availability statement

The data presented in the study are deposited in the NCBI repository (https://www.ncbi.nlm.nih.gov/sra), accession number PRJNA890171.

## Ethics statement

The studies involving human participants were reviewed and approved by Medical Ethics Committee of the Women and Children’s Hospital, School of Medicine, Xiamen University. The patients/participants provided their written informed consent to participate in this study.

## Author contributions

CT and XZ contributed to design the study, research data and wrote the manuscript. JX and SH contributed to data interpretation and the discussion of the results. YTX contributed to methodology. JZ and YL contributed to design the study and critically reviewed the manuscript. YSX, QW, and LM contributed to subject recruitment, sample collection, and perform clinical examination. HS conceptualized and designed the protocol and critically reviewed the manuscript. All authors contributed to the article and approved the submitted version.
