# Supplementary figures and images for: Maternal vaginal fluids play a major role in the colonization of the neonatal intestinal microbiota
Source: Front Cell Infect Microbiol. 2023 Mar 15;13:1065884. doi: 10.3389/fcimb.2023.1065884 (PMC10061231; doi:10.3389/fcimb.2023.1065884)

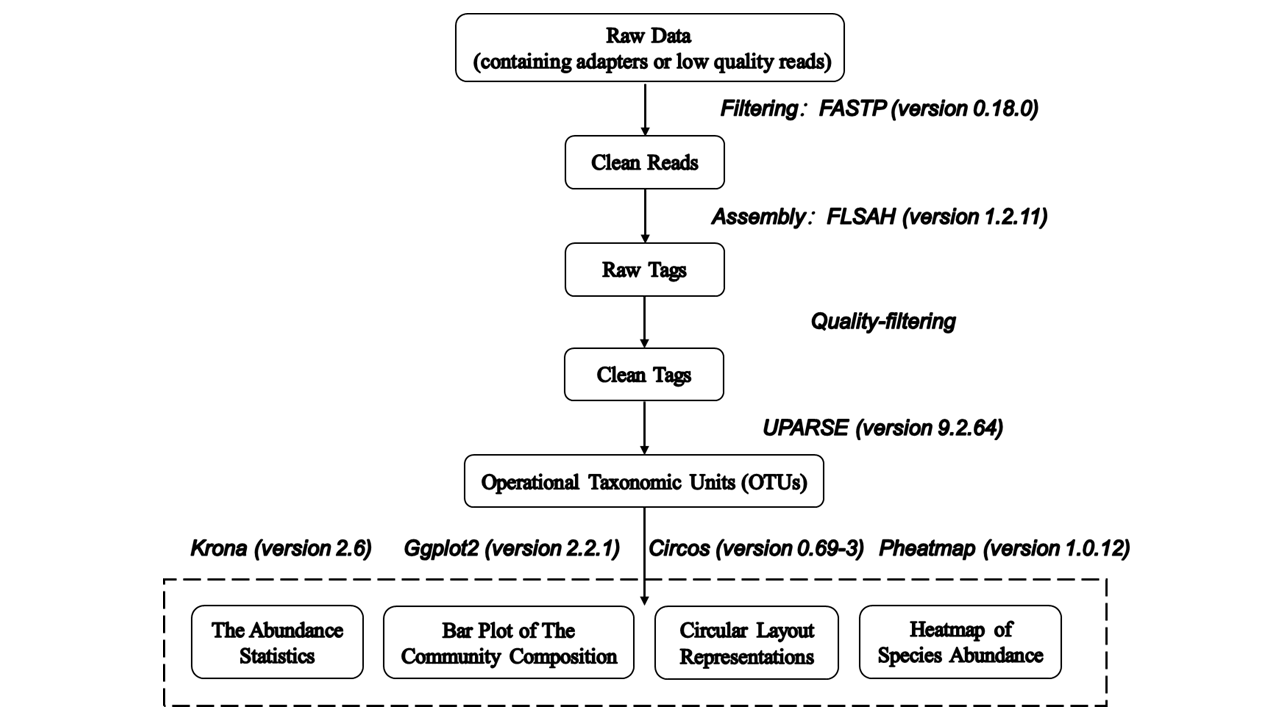

Supplement: Supplementary file 1 [file Image_1.tif]

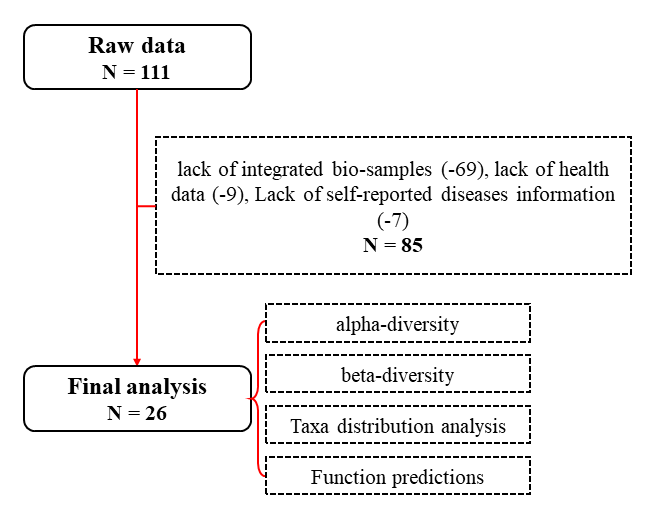

Supplement: Supplementary file 2 [file Image_2.tif]

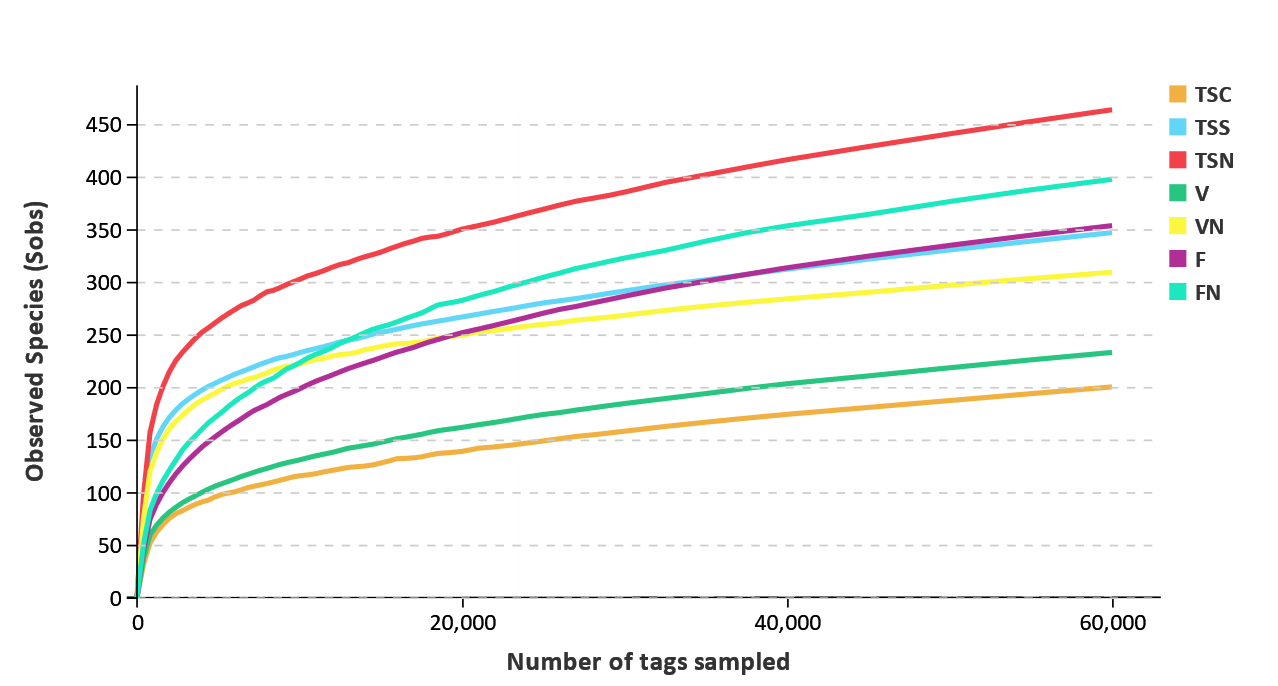

Supplement: Supplementary file 3 [file Image_3.tif]
